# Supplementary figures and images for: The pcnB gene sustains Shigella flexneri virulence
Source: PLoS Pathog. 2025 Nov 20;21(11):e1013727. doi: 10.1371/journal.ppat.1013727 (PMC12779154; doi:10.1371/journal.ppat.1013727)

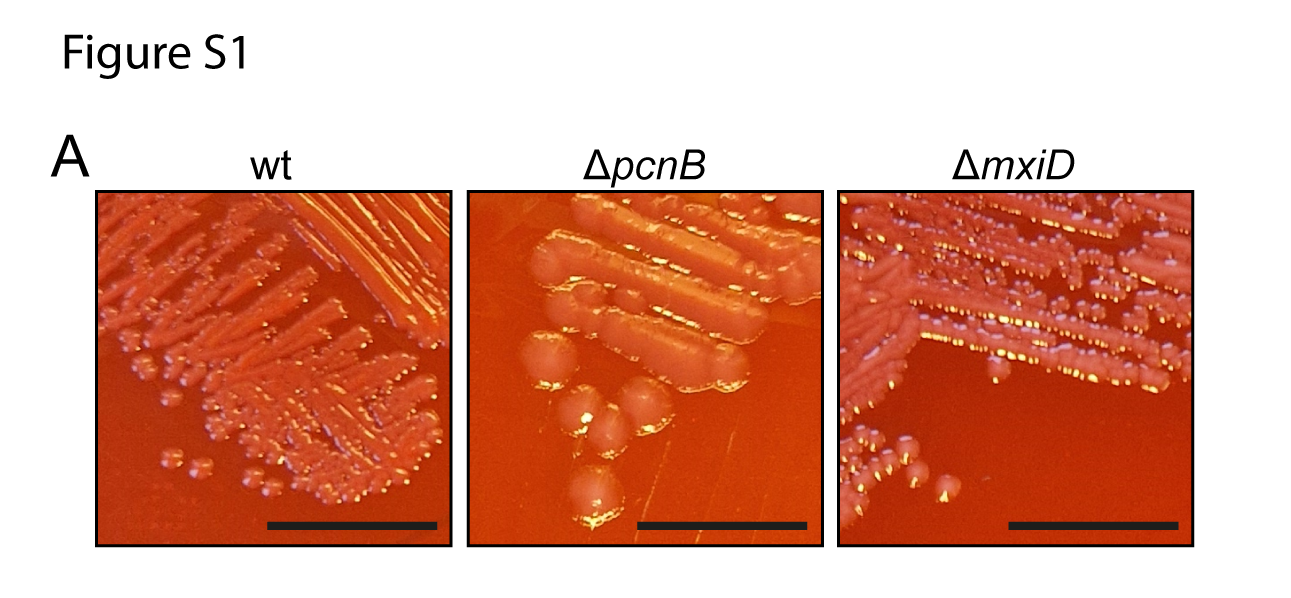

Supplement: S1 Fig — (A) Representative images of CR binding of Shigella wt, ΔpcnB and ΔmxiD strains grown at 37°C ON. The three strains were streaked on the same CR plate. Scale bar: 10mm. (TIF) [file ppat.1013727.s001.tif]

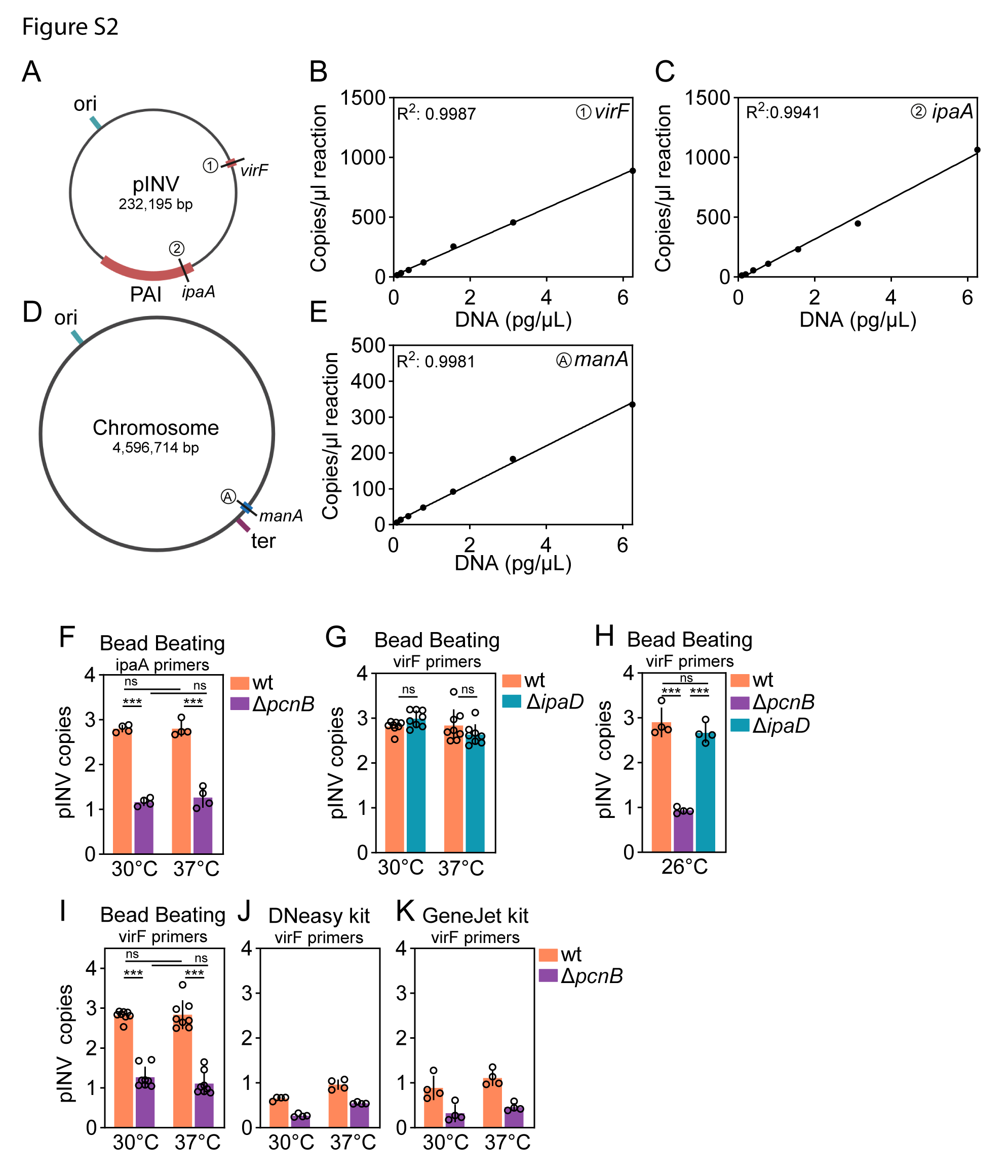

Supplement: S2 Fig — (A) Schematic representation of Shigella M90T pINV [6]. Ori is indicated in teal. PAI and virF position are indicated in red. Position of primer pairs used to detect pINV are indicated by black lines. Partially made with BioRender. (B) Standard curve for ddPCR detection of pINV using primer pair 1 targeting the virF gene (as in S2A Fig; see S2 Table). 6.25 pg/µl of gDNA extracted by bead beating from Shigella wt were diluted in two-fold serial dilutions (up to 0.097656 pg/µl) and used to generate standard curve for virF primer pair. R2 value indicated in the top left corner of the panel. (C) Standard curve for ddPCR detection of pINV using primer pair 2 targeting the ipaA gene (as in S2A Fig; see S2 Table). 6.25 pg/µl of gDNA extracted by bead beating from Shigella wt were diluted in two-fold serial dilutions (up to 0.097656 pg/µl) and used to generate standard curve for ipaA primer pair. R2 value indicated in the top left corner of the panel. (D) Schematic representation of Shigella M90T chromosome. Ori is indicated in teal; ter region is indicated in purple. manA gene position is indicated in blue. Position of primer pair used to detect the chromosome is indicated by black line. Partially made with BioRender. (E) Standard curve for ddPCR detection of Shigella chromosome using primer pair A targeting the manA gene (as in S2D Fig; see S2 Table). 6.25 pg/µl of gDNA extracted by bead beating from Shigella wt were diluted in two-fold serial dilutions (up to 0.097656 pg/µl) and used to generate standard curves for manA primer pair. R2 value indicated in the top left corner of the panel. (F) pINV relative copies per chromosome was determined by ddPCR using ipaA primers for pINV and manA primers for chromosome. gDNA was extracted by bead beating from Shigella wt or ΔpcnB mutant grown at 30°C or 37°C at OD600 0.7. Data comes from 8 biological replicates from two independent experiments. Statistical significance determined by Two-way Anova; ns - non-significant; ***p < 0.00 [file ppat.1013727.s002.tif]

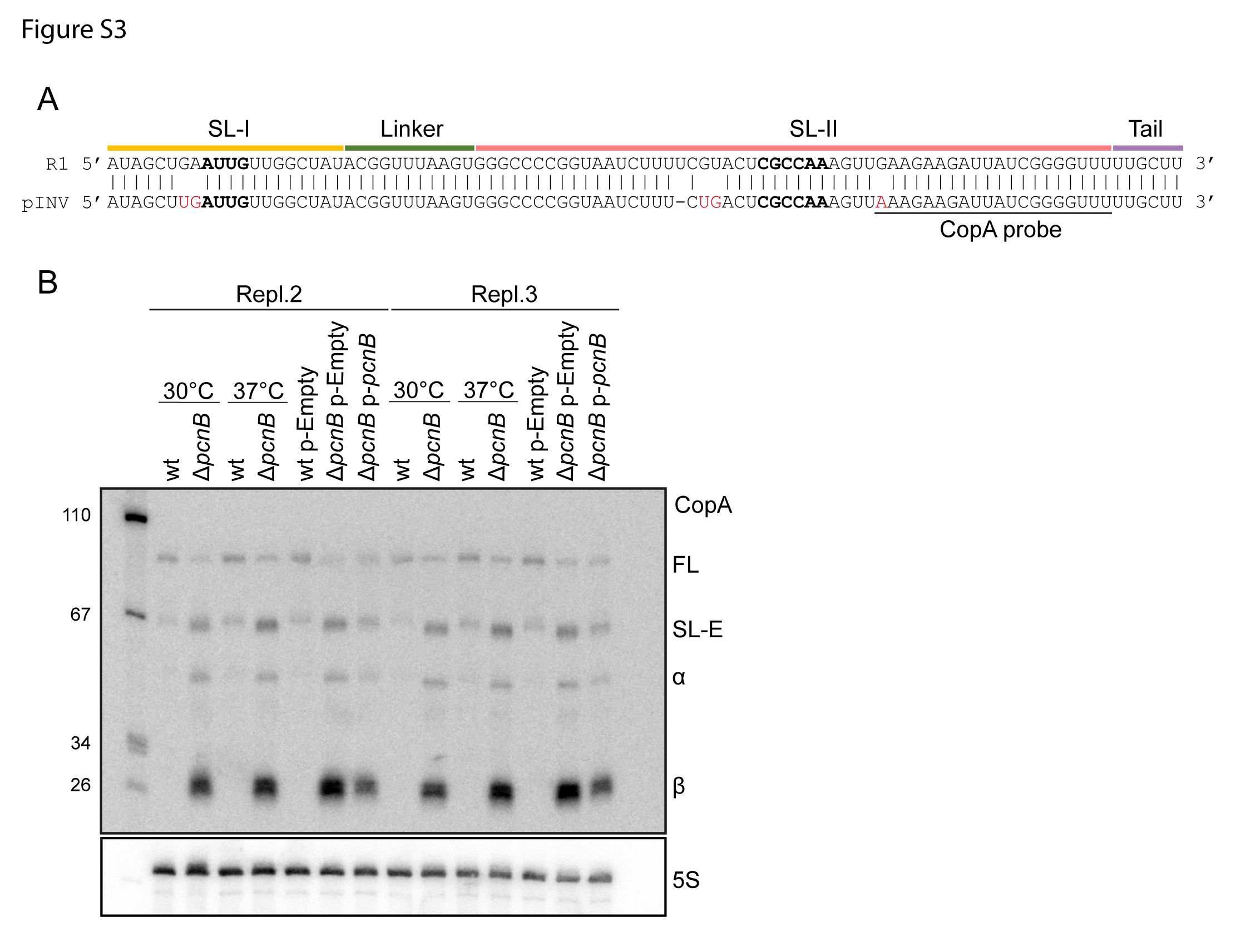

Supplement: S3 Fig — (A) Sequence alignment between CopA from R1 plasmid and CopA from Shigella pINV. Nucleotide changes are indicated in red. Sequences for the bulge of stem-loop I and stem-loop II are indicated in bold and refer to CopA-R1 secondary structure [86]. (B) Replicates 2 and 3 of quantitative northern blot of CopA species in Shigella wt or ΔpcnB mutant grown at 30°C or 37°C at OD600 0.7 and in Shigella wt or ΔpcnB strains carrying the p-Empty or p-pcnB plasmid, grown at 37°C at OD600 0.7. (TIF) [file ppat.1013727.s003.tif]

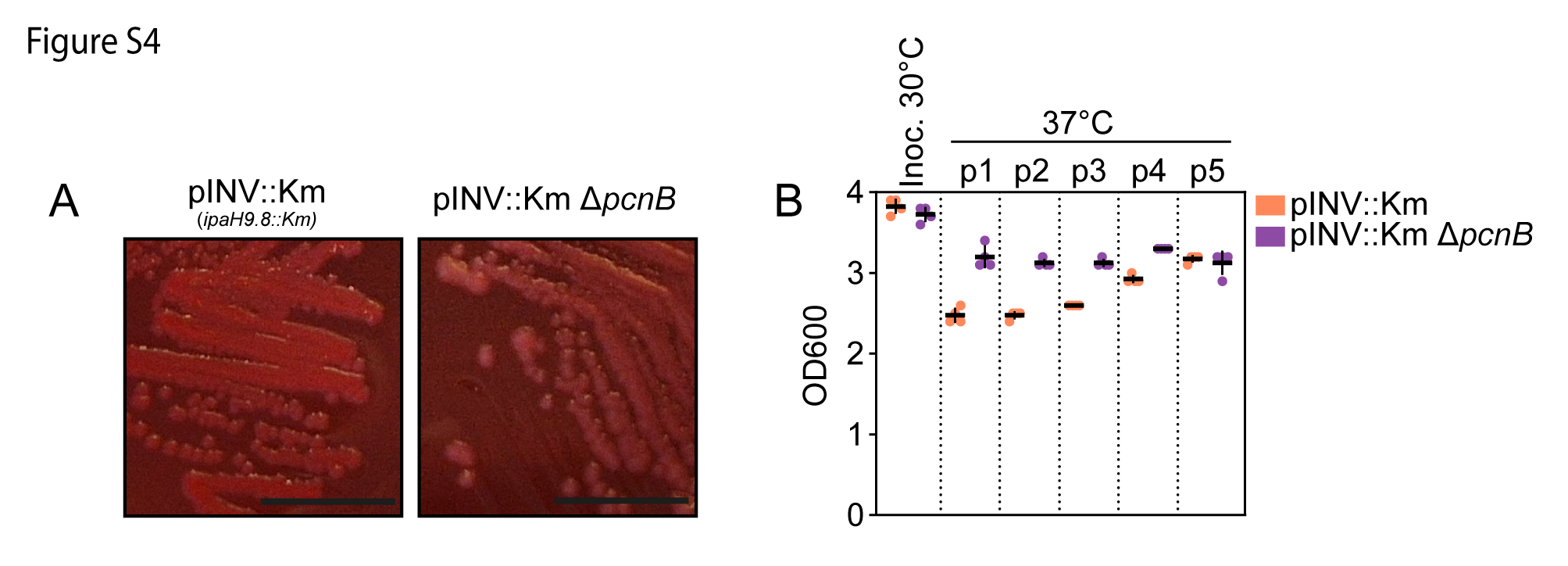

Supplement: S4 Fig — (A) Representative images of CR binding of Shigella pINV::Km (ΔipaH9.8) and pINV::Km ΔpcnB strains grown at 37°C ON. The two strains were streaked on the same CR plate. Scale bar: 10mm. (B) OD600 measurement of pINV::Km and pINV::Km ΔpcnB overnight (ON) cultures grown at 30°C (inoculum) and thereafter passaged daily at 37°C. (TIF) [file ppat.1013727.s004.tif]
