# Supplementary material for: The pcnB gene sustains Shigella flexneri virulence
Source: PLoS Pathog. 2025 Nov 20;21(11):e1013727. doi: 10.1371/journal.ppat.1013727 (PMC12779154; doi:10.1371/journal.ppat.1013727)
Supplement: Attachment — Submitted filename: Frisch et al_Response to reviewers.pdf [file ppat.1013727.s009.pdf]

Uppsala, 02 October 2025

We would like to thank all three reviewers for their positive evaluations and constructive suggestions on our manuscript PPATHOGENS-D-25-00628 entitled "The *pcnB* gene sustains *Shigella flexneri* virulence". We have incorporated these suggestions and responded point-by-point to each comment below. The original reviewer comments are included in *italicized* text, followed by our replies. In addition to clarifications in writing, we have conducted several additional experiments, guided by the reviewer comments. This has led to the incorporation of new experimental data and analyses in the revised manuscript, specifically:

I. A new main Figure that assesses *pcnB* impact on *Shigella* virulence plasmid stability (Fig 5).

II. A new supplementary figure with supporting data for Fig 5 (Fig S4).

III. New *Shigella* enteroid infection assays to further confirm the role of *pcnB* in sustaining *Shigella* intraepithelial expansion (new data included in Fig 6, panels D-E).

IV. A revised quantification of FL-CopA and SL-E CopA antisense RNA forms from northern blots to better circumstantiate their collective role in pINV replication control (new analyses included in Fig 4, panels C-D).

Taken together, these revision experiments and analyses have furthered strengthened the conclusions of our study and provided important context. We have adapted the manuscript text accordingly, as specified below. Please note that the figure call outs and line numbers reported in this response letter refer to the .docx version of the revised manuscript.

<u>*Part I - Summary*</u>
Please use this section to discuss strengths/weaknesses of study, novelty/significance, general execution and scholarship.

<u>*Reviewer #1:*</u>

In this manuscript, Frisch and colleagues characterize the involvement of the pcnB gene in the virulence of Shigella flexneri, the etiologic agent responsible for bacillary dysentery. They show that pcnB facilitates the expression of virulence determinants, specifically those belonging to the Type III Secretion System (T3SS), which are encoded on the virulence plasmid pINV. The authors also demonstrate that pcnB helps maintain optimal pINV copy number through the degradation of the antisense RNA CopA, the principal regulator of plasmid replication. pcnB-mediated equilibrium between bacterial proliferation and virulence expression allows Shigella flexneri to invasively colonize and disseminate in human intestinal epithelial cells efficiently, as shown in this work using enteroid models.

Overall, although the basis of pcnB function and the control of plasmid replication by antisense RNA has been known for years in other model systems, this study provides a comprehensive physiological demonstration of the role of pcnB in pINV-mediated Shigella virulence. The combination of molecular analyses (plasmid copy number and CopA RNA stability), global proteomic data, and functional validation using an advanced human infection model (enterocytes) distinguishes this work from existing data and gives it good impact in the field of bacterial pathogenesis. Previously published or preliminary data cited serve as a starting or supporting point, reinforcing the relevance of this investigation.

Throughout the manuscript, data are well presented, in general, it is well written, and figures are informative and clear. The approaches used in this study are sound. The quality of the data

is good, and the use of statistics is appropriate. The methodologies used are very well described, with details to reproduce the work. References are appropriate to credit previous works.

Response: We are happy to learn of this positive overall assessment. Below we specify how the remaining concerns of reviewer #1 have been addressed.

<u>Reviewer #2:</u>

In this report, Frish et al. show that the chromosomal gene pcnB sustains an optimal virulence plasmid copy number in Shigella flexneri. The results demonstrate that plasmid copy number is critical for optimal T3SS protein expression, with a significant impact on invasion, replication and cell-to-cell spread within epithelial cells. The work is of high quality and the conclusions are solid. This reviewer has only minor comments that involve serious editing. One additional experiment is also suggested.

Response: We thank reviewer #2 for these positive remarks.

<u>Reviewer #3:</u>

•Overall, this is a well-organized, logical study.

•The main finding of this study is that PcnB influences pINV copy number via alteration of active CopA levels. All other observations are the logical consequences of this regulation. Furthermore, there are no investigations into the molecular mechanism mediating PcnB regulation of CopA activity. As a result, the impact of this study is limited.

•Significance of the findings is overstated, specifically in the claim that PcnB functions to maintain "optimal levels of pINV".

Response: We thank reviewer #3 for the constructive remarks, which have been considered as specified below. We would here like to emphasize that we have indeed formally shown that *pcnB* sustains pINV replication via alteration of active CopA levels, as previously characterized for plasmids harbouring the same origin of replication (i.e. R1 plasmids). However, we believe that the central novelty of our work, as highlighted also by reviewer #1 above, lies in providing a comprehensive physiological understanding of the impact of *pcnB* on *Shigella* virulence. In particular, our present study: i) following a genome-wide Tn-Seq screen, substantiates the importance of the *pcnB* gene in promoting *Shigella* colonization of a physiologically relevant human enteroid infection model; ii) presents comprehensive data on the p*cnB*-promoting effect on global virulence protein expression; iii) shows that *pcnB* sustains pINV virulence plasmid replication; iv) provides formal mechanistic evidence on the *pcnB* role in CopA degradation in this context and, v) elucidates how the resulting pINV level is critical for maximizing *Shigella* intraepithelial expansion. After revision, we have also included a new Figure (Fig 5), demonstrating that *pcnB* does not affect pINV stability.

<u>Part II – Major Issues: Key Experiments Required for Acceptance</u>
Please use this section to detail the key new experiments or modifications of existing experiments that should be <u>absolutely</u> required to validate study conclusions.

Generally, there should be no more than 3 such required experiments or major modifications

for a "Major Revision" recommendation. If more than 3 experiments are necessary to validate the study conclusions, then you are encouraged to recommend "Reject".

Reviewer #1:

1. The authors state that formally assessing whether pcnB affects the stability (i.e., loss) of the pINV plasmid, in addition to its replication control, is complicated by potential confounding factors such as pINV chromosomal integration and its high genetic plasticity. They conclude that pINV loss in the ΔpcnB mutant is negligible under the tested conditions, based on the restoration of plasmid copy number upon pcnB complementation. Could the authors elaborate on the rationale for considering plasmid loss negligible based solely on complementation data? Would it be feasible to design a dedicated experiment to directly quantify plasmid loss in the absence of pcnB under specific conditions (e.g., prolonged growth or absence of selective pressure for the plasmid)? Such data could more formally distinguish effects on replication from those on plasmid stability.

Response:

We thank the reviewer for the insightful comment and the opportunity to further elaborate on plasmid stability. The *Shigella* virulence plasmid (pINV) encodes two partitioning systems and three toxin-antitoxin (TA) modules, which collectively contribute to its maintenance under various growth conditions (1, 2). Previous studies have shown that at 37°C, where virulence gene expression is active and selection pressure therefore is present, the TA system MvpTA ensures plasmid retention for at least 50 generations (1). However, despite plasmid maintenance, over 50% of the population exhibits a Congo Red (CR) negative phenotype at this point, indicating loss of virulence. This phenomenon was primarily attributed to the selective deletion of key virulence loci, notably the transcriptional regulator genes *virF* and *virB* or the entire Entry region, which can be lost in the majority of the population by generation 50. Thus, while the plasmid remains present, *Shigella* progressively becomes avirulent when cultured at 37°C (1).

The CR phenotype has served as a valuable proxy for assessing virulence loss and plasmid stability in *Shigella* (1, 3). However, the Δ*pcnB* mutant already displays a CR^- phenotype (see Fig S1 and Fig S4A), complicating the use of this readout in experimental setups. As the reviewer pointed out, we suggested that pINV loss in the Δ*pcnB* mutant is minimal under the tested conditions. This inference is based on the successful restoration of *pcnB* expression and plasmid copy number following *pcnB* complementation (Fig 3D-E). We reasoned that if pINV was rapidly lost in the majority of the population (within 10–15 generations), transformation with the *pcnB*-expressing plasmid would likely fail to restore plasmid copy number. We acknowledge, however, that this argument may appear speculative.

Inspired by the reviewer's comment, we sought an alternative strategy to assess the impact of *pcnB* deletion on pINV stability, which resulted in the addition of a new main figure (Fig 5) and a new supplementary figure with supporting data (Fig S4). Due to repeated unsuccessful attempts to introduce an antibiotic resistance cassette directly into an inert location on pINV in the *Shigella* Δ*pcnB* mutant background, we instead introduced the *pcnB* deletion into an *ipaH9.8* mutant strain. In this strain, the *ipaH9.8* gene, located on pINV, is disrupted by a kanamycin resistance cassette. The *ipaH9.8* mutant retains a CR^+ phenotype (Fig S4A) and, as previously shown, does not impair *Shigella* invasion or intraepithelial expansion in an enteroid infection model (4). It can therefore be considered a suitable control strain, behaving comparably to wild-type during infection while providing a selectable marker on pINV. We grew this control strain (hereafter pINV::Km) alongside the pINV::KmΔ*pcnB* mutant strain over 50 generations at 37°C, when virulence gene expression is active, and we assessed plasmid stability over time. The results of these new experiments show that *pcnB* does not impact pINV plasmid stability or maintenance under physiological conditions. As previously observed, the

control strain progressively accumulates CR- avirulent colonies due to selective loss of key virulence loci such as *virB*, *virF*, or others. Notably, this loss was not detected in the Δ*pcnB* mutant, suggesting that reduced plasmid copy number in this strain may lower the selective pressure driving plasmid-encoded virulence gene loss. While we cannot exclude a potential role for *pcnB* in plasmid loss or stability in the absence of active partitioning and toxin-antitoxin (TA) systems, our data indicate that under physiological conditions, where TA systems are functional, *pcnB* does not affect pINV stability (see Fig 5).

2. The data presented in this manuscript show that pcnB complementation restores degradation of the processed CopA-SL-E form to wild-type levels but does not restore the relative abundance of the full-length CopA-FL transcript (Figure 4). The authors suggest that CopA-SL-E is sufficient for replication control. Given that CopA-FL persists at elevated levels in the ΔpcnB mutant and is not fully restored by complementation, could this have additional effects not captured by the current assays (e.g., impacts on Tap/RepA translation beyond CopA-SL-E degradation, or other RNA interactions)? Could there be additional mechanisms regulating CopA-FL abundance or function that are independent of pcnB and PAP-I, or residual degradation activity in the mutant not enhanced by pcnB? A brief discussion on the physiological significance of the incomplete restoration of CopA-FL would be helpful.

Response: After rereading the relevant manuscript passages with this comment in mind, we agree that this point requires clarification. The *Shigella* pINV virulence plasmid contains an IncFII origin of replication, similar to the well-characterized *Salmonella* R1 plasmid, which has served as a model system for studying plasmid replication control (5). Prior work on the R1 plasmid has shown that both CopA-FL and CopA-SL-E contribute to inhibition of RepA translation (6). Since CopA is known to be constitutively expressed, the observed reduction in CopA-FL levels in the *Shigella* Δ*pcnB* mutant reflects a decrease in plasmid copy number. Conversely, CopA-SL-E levels increase in the Δ*pcnB* mutant due to reduced degradation and consequently increased transcript half-life. Therefore, the relative proportions of these two forms differ markedly between the two strains at both 30° and 37°C. To better illustrate these dynamics, we have now revised the presentation of the quantitative data from the northern blot analysis (Fig 4C-D). Moreover, the revised data presentation also highlights that although *pcnB* complementation does not fully restore the relative abundance of CopA-FL to wild-type levels, the total amount of CopA transcripts (FL + SL-E), both functional in replication control, matches that of the wild-type strain (Fig 4D). This explains the restoration of pINV copy number to wild-type levels upon *pcnB* complementation (Fig 3E). We have clarified this aspect in the corresponding manuscript section (Lines 239-243). Taken together, our data provide strong and formal support for a conserved mechanism of plasmid replication control in *Shigella* pINV, closely resembling that of the R1 plasmid.

3. The discussion mentions that polyadenylation by PAP-I creates a platform for 3' exonucleases (PNPase and RNase II) to initiate degradation after RNase E cleavage. It is also noted that pnp is required for colonization. It would be informative to experimentally test the involvement of PNPase and/or RNase II in pcnB-mediated accelerated degradation of CopA, particularly the CopA-SL-E form (for instance, by examining CopA stability in pnp or rnb (RNase II) mutants, alone or in combination with the ΔpcnB mutant).

Response: We appreciate the reviewer's insightful comment and fully agree that it raises a relevant point. The degradation of CopA by the exoribonucleases PNPase and RNase II has been systematically studied in the context of the R1 plasmid (7). Specifically, it was shown that: i) both PNPase and RNase II can degrade SL-E; ii) these enzymes can functionally compensate for each other, since single deletions have only a minor impact on SL-E half-life; iii) both require PcnB activity to promote SL-E decay; and iv) the presence of RNase II reduces the rate of PNPase-dependent SL-E degradation, suggesting competition at the 3′ end of the CopA SL-E transcript.

In summary, CopA decay is initiated by RNase E, followed by PcnB-mediated polyadenylation of SL-E, which is a prerequisite for efficient 3′-exonucleolytic degradation by PNPase and/or RNase II (7).

Given that pINV replication control faithfully mirrors the regulatory mechanisms of the R1 plasmid (as shown in Fig 4) we chose not to replicate these well-established degradation experiments, since this would add little novelty to the current manuscript. Instead, considering the infection biology focus of our study, we prioritized expanding and strengthening the characterization of the *Shigella* intraepithelial expansion phenotype during colonization of intestinal epithelial organoids, as presented in Fig 6D-E.

Reviewer #2:

NA

Response: We are happy to learn that reviewer #2 does not report any major issues.

Reviewer #3:

1. Is the impact of PcnB on pINV copy number mediated exclusively via regulation of CopA? ie: Are PcnB-mediated alterations observed in a strain lacking CopA?

Response: This comment gives us the opportunity to further clarify the organization and regulatory significance of the RepFIIA replicon. The *Shigella* pINV virulence plasmid contains a RepFIIA replicon, similar to the well characterized *Salmonella* R1 plasmid, which has served as a model system for understanding plasmid replication control (5, 8). The replication region of R1 is organized into a tightly regulated operon that ensures low plasmid copy number and stable inheritance (see Fig R1 in this rebuttal). At the core of this system is RepA, the initiator protein that binds directly to the plasmid's origin of replication (ori), enabling the initiation of DNA replication. Its expression is finely controlled at both transcriptional and post-transcriptional levels by CopB and CopA, respectively. CopB is a DNA-binding protein that represses the strong p*repA* promoter, modulating *repA* transcription in response to plasmid copy number. CopA, located downstream of *copB*, encodes a small antisense RNA transcribed from the opposite strand of *repA*. It binds to the complementary CopT sequence in the 5′ UTR of *repA* mRNA, blocking translation and promoting degradation via RNase III. Specifically, CopA interferes with translation of the upstream Tap peptide, which is essential for efficient RepA translation due to translational coupling.

This multi-layered regulation is made possible by the rigid genetic organization of the replication region. Importantly, because CopA is transcribed antisense to *repA*, deleting CopA would also disrupt the CopT region, directly impairing *repA* mRNA structure and regulation. Previous studies have shown that loss of CopA function (i.e. impaired transcription) leads to uncontrolled plasmid replication, a phenomenon known as runaway replication, which is lethal to the host (5). Based on these previous observations, a strain lacking CopA will suffer from plasmid instability and compromised host viability. This genetic wiring makes it impossible to in a meaningful way assess effects of PcnB on pINV copy number in the presence versus absence of CopA.

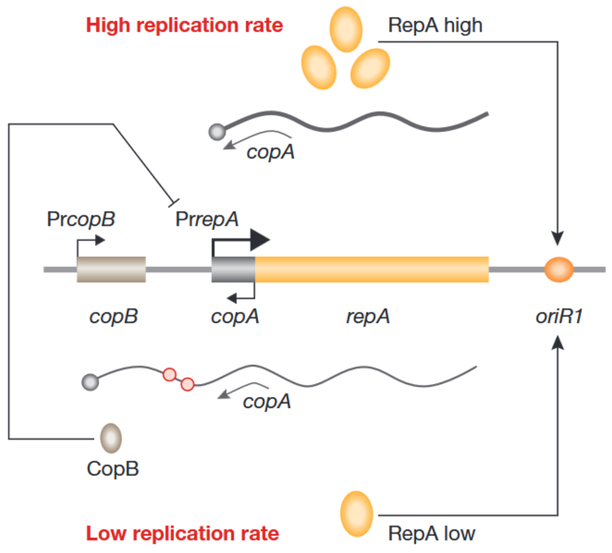

Figure R1. Schematic representation of the basic R1 replicon. The basic replicon of plasmid R1 includes: the origin of replication (oriR1); the *repA* gene for the initiator protein RepA; the *tap* gene for a leader peptide needed for RepA synthesis (in grey); *copA*, which encodes an antisense RNA that inhibits *repA* translation; *copB*, which encodes a repressor of the prepA promoter; and the constitutive promoters Pr*copA* and Pr*copB*. Figure from (8)

2.How does PcnB modulate CopA levels/activity?

Response: PcnB encodes the poly-A polymerase PAP-I, an enzyme responsible for adding poly-A tails to the 3′ ends of numerous bacterial transcripts, a modification typically associated with RNA degradation (9, 10). Previous studies have linked mutations in *pcnB* to the accumulation of RNase E-dependent cleavage products of the antisense RNA CopA (see Figure 4), which plays a central role in regulating replication of the R1 plasmid (6) (see also response to comment 1). CopA is constitutively transcribed, but undergoes rapid degradation to maintain stringent control over plasmid replication. This decay is initiated by RNase E and followed by PcnB-mediated polyadenylation of the RNase E-dependent cleavage fragment SL-E. In turn, polyadenylation facilitates efficient 3′-exonucleolytic degradation by PNPase and/or RNase II (6, 7). In wild-type strains (*pcnB*$^{+}$), the SL-E fragment is rapidly degraded. However, in Δ*pcnB* mutant strains, SL-E accumulates due to impaired degradation, resulting in increased transcript stability. Analogous to for the R1 plasmid, our data demonstrate that *pcnB* supports *Shigella* pINV plasmid copy number by promoting the decay of the antisense RNA CopA, which inhibits RepA translation (see Figure 4). We have modified the text to more clearly spell out how PcnB modulates CopA levels (Lines 217-219, Lines 231-232, Lines 239-243).

3.The conclusion is drawn that PcnB enhances Shigella virulence by "sustaining Shigella pINV replication to this optimal level". This statement implies some sort of safe-guard against too few or too many copies of pINV. The study demonstrates that without PcnB, pINV copy number decreases, it says nothing about preventing too many copies of the plasmid.

Response: We appreciate the reviewer's thoughtful observation. Our statement regarding PcnB "sustaining *Shigella* pINV replication to this optimal level" was intended to reflect the idea that PcnB contributes to achieve a plasmid copy number that supports full virulence expression. We agree that our data specifically demonstrate a decrease in pINV copy number in the absence of PcnB, and do not directly address the possibility of excessive plasmid replication.

The concept of an "optimal" pINV level was based on the functional consequences observed in the *Shigella* wt strain, where the steady-state copy number appears sufficient to support robust expression of pINV-encoded virulence factors, albeit with a mild fitness cost. This interpretation is supported by the tight regulation of IncFII plasmid replication, as documented for the R1 plasmid, where deviations from the steady-state are typically detrimental (5). In the context of *Shigella*, the reduced pINV copy number in the Δ*pcnB* mutant correlates with increased fitness under virulence inducing conditions (Fig 1A-C), lowered abundance of key

virulence proteins (Fig 2) and impaired intraepithelial expansion during infection of intestinal organoid-derived monolayers (Fig 6).

This again highlights the concept of a fitness–virulence tradeoff, where maintaining a plasmid copy number that ensures virulence factor expression comes at a metabolic cost, but is nonetheless beneficial for successful infection. We have revised the text to avoid implying a regulatory safeguard against excessive pINV replication, and instead emphasize the role of PcnB in supporting a pINV level suitable for *Shigella* virulence.

Part III – Minor Issues: Editorial and Data Presentation Modifications
Please use this section for editorial suggestions as well as relatively minor modifications of existing data that would enhance clarity.

Reviewer #1:

4. The reviewer appreciates the clear definition of the statistical significance criteria used for the proteomic analysis (|Log2FC| ≥ 0.5; adj_p_value ≤ 0.01). Could the authors provide further details on the scientific rationale behind the choice of the Log2FC threshold at 0.5, which is quite low? Were alternative thresholds (such as Log2FC ≥ 1.0) considered or evaluated, and if so, how would the results—in terms of the number and identity of differentially expressed proteins (particularly those encoded on pINV)—and their biological interpretation have changed?

Response: While a Log_2FC threshold of 1.0 (equivalent to a 2-fold change) is commonly used in proteomic analyses for general comparisons, it is important to consider that biological systems often rely on coordinated regulation, where even modest changes in protein abundance can have significant phenotypic consequences. This is particularly relevant in our study, where many of the proteins showing lower abundance in the Δ*pcnB* mutant are encoded within the same regulons or operons, such as those located on the pINV plasmid.

As detailed in Table R1, applying a Log_2FC threshold of 1.0 identifies 21 significantly less abundant pINV-encoded proteins in the *Shigella* Δ*pcnB* mutant compared to wild type. Lowering the threshold to 0.5 reveals 57 proteins with reduced abundance. Notably, both thresholds capture key components of the T3SS machinery and effectors encoded by the ipa-ipg and mxi-spa operons. These operons are located in the pINV entry region and are known to be transcriptionally co-regulated via the VirF-VirB virulence cascade (11) (see Fig R2). Therefore, the consistently lower abundance of these functionally related proteins, even at Log_2FC values below 1, suggests a biologically relevant shift. This interpretation is further supported by the robustness of our dataset, which includes four biological replicates per strain (see also response to comment #5).

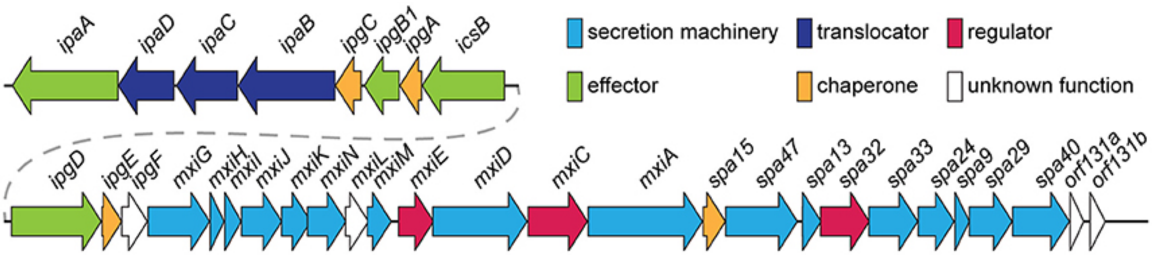

Figure R2. Entry region of pINV, organized into two operons (*ipa/ipg* and *mxi/spa*) encoding T3SS components and effectors. Genes are color-coded by function. Figure from Mattock et al. 2017.

	protein_id	locus_tag	com_id	description	comparisc	log2_fc	adj_p_valu
1	QCC34415.1	EKN05_024725	MxiH	type III secretion system needle complex protein	pcnB-WT	-2.96164	6.06E-05
2	QCC34355.1	EKN05_024195	YigA	ANR family transcriptional regulator	pcnB-WT	-2.57262	0.000105
3	QCC34452.1	EKN05_023905	IpgB2	hypothetical protein	pcnB-WT	-1.76703	1.6E-05
4	QCC34387.1	EKN05_024500	IcsA	autotransporter outer membrane beta-barrel domain-containing protein	pcnB-WT	-1.46971	1.97E-06
5	QCC34340.1	EKN05_024040	PhoN2	phosphatase PAP2 family protein	pcnB-WT	-1.40856	4.29E-06
6	QCC34424.1	EKN05_024775	IpaB	type III secretion system needle tip complex protein SipB	pcnB-WT	-1.35728	2.45E-05
7	QCC34454.1	EKN05_024070	IcsP	omptin family outer membrane protease IcsP	pcnB-WT	-1.30678	2.82E-06
8	QCC34336.1	EKN05_024000	OspF	type III secretion system effector phosphothreonine lyase	pcnB-WT	-1.25561	7.94E-07
9	QCC34429.1	EKN05_024800	VirB	Virulence regulon transcriptional activator VirB	pcnB-WT	-1.23144	1.21E-06
10	QCC34420.1	EKN05_024755	IcsB	Virulence protein IcsB	pcnB-WT	-1.17138	7.94E-07
11	QCC34337.1	EKN05_024005	OspD2	ShET2/EspL2 family type III secretion system effector toxin	pcnB-WT	-1.1593	0.000149
12	QCC34412.1	EKN05_024705	MxiN	Putative membrane protein	pcnB-WT	-1.15796	1.21E-06
13	QCC34411.1	EKN05_024700	MxiL	Mxi-Spa secretion machinery protein MxiL	pcnB-WT	-1.13225	0.000282
14	QCC34383.1	EKN05_024445	PWR501_(	type II toxin-antitoxin system RelB/DinJ family antitoxin	pcnB-WT	-1.12225	0.00333
15	QCC34425.1	EKN05_024780	IpaC	IpaC/SipC family type III secretion system effector	pcnB-WT	-1.08006	7.94E-07
16	QCC34335.1	EKN05_023985	Orf13	hypothetical protein	pcnB-WT	-1.06787	4.14E-06
17	QCC34427.1	EKN05_024790	IpaA	invasin IpaA	pcnB-WT	-1.0565	7.94E-07
18	QCC34404.1	EKN05_024665	Spa47	FliI/YscN family ATPase	pcnB-WT	-1.05568	1.21E-06
19	QCC34417.1	EKN05_024735	IpgF	protein IpgF	pcnB-WT	-1.04965	1.21E-06
20	QCC34426.1	EKN05_024785	IpaD	type III secretion system needle tip complex protein IpaD	pcnB-WT	-1.02536	8.47E-07
21	QCC34308.1	EKN05_023710	OspC2	hypothetical protein	pcnB-WT	-1.02393	0.000133
22	QCC34407.1	EKN05_024680	MxiC	YopN family type III secretion system gatekeeper subunit	pcnB-WT	-0.97853	4.26E-06
23	QCC34419.1	EKN05_024745	IpgD	type III secretion system effector inositol phosphate phosphatase	pcnB-WT	-0.96726	1.6E-06
24	QCC34462.1	EKN05_024710	MxiK	type III secretion apparatus protein OrgA/MxiK	pcnB-WT	-0.95234	0.001106
25	QCC34403.1	EKN05_024660	Spa13	surface presentation of antigens protein SpaM	pcnB-WT	-0.9406	0.000763
26	QCC34396.1	EKN05_024625	Spa-orf10	hypothetical protein	pcnB-WT	-0.93864	6.91E-05
27	QCC34331.1	EKN05_023925	OspD1	hypothetical protein	pcnB-WT	-0.93121	3.61E-06
28	QCC34434.1	EKN05_024840	OspC3	hypothetical protein	pcnB-WT	-0.92748	2.81E-05
29	QCC34388.1	EKN05_024505	VirA	cysteine protease	pcnB-WT	-0.92322	4.26E-06
30	QCC34430.1	EKN05_024805	IpaJ	type III secretion system effector cysteine protease IpaJ	pcnB-WT	-0.9207	2.82E-06
31	QCC34408.1	EKN05_024685	MxiD	EscC/YscC/HrcC family type III secretion system outer membrane ring protein	pcnB-WT	-0.89239	1.32E-06
32	QCC34406.1	EKN05_024675	MxiA	EscV/YscV/HrcV family type III secretion system export apparatus protein	pcnB-WT	-0.88258	2.82E-06
33	QCC34400.1	EKN05_024645	Spa24	EscR/YscR/HrcR family type III secretion system export apparatus protein	pcnB-WT	-0.87144	9.12E-05
34	QCC34413.1	EKN05_024715	MxiJ	EscJ/YscJ/HrcJ family type III secretion inner membrane ring protein	pcnB-WT	-0.86798	1.84E-06
35	QCC34410.1	EKN05_024695	MxiM	AraC family transcriptional activator MxiE	pcnB-WT	-0.85486	1.43E-06
36	QCC34416.1	EKN05_024730	MxiG	protein MxiG	pcnB-WT	-0.85397	0.000125
37	QCC34418.1	EKN05_024740	IpgE	type III secretion system chaperone	pcnB-WT	-0.85359	3.3E-06
38	QCC34357.1	EKN05_024205	TraX	type-F conjugative transfer system pilin acetylase TraX	pcnB-WT	-0.84124	0.000124
39	QCC34421.1	EKN05_024760	IpgA	chaperone protein IpgA	pcnB-WT	-0.83399	3.33E-06
40	QCC34401.1	EKN05_024650	Spa33	surface presentation of antigens protein SpaO	pcnB-WT	-0.80519	0.002397
41	QCC34423.1	EKN05_024770	IpgC	type III secretion system translocator chaperone SicA	pcnB-WT	-0.79237	1.98E-05
42	QCC34409.1	EKN05_024690	MxiE	helix-turn-helix domain-containing protein	pcnB-WT	-0.78471	1.03E-05
43	QCC34402.1	EKN05_024655	Spa32	surface presentation of antigens protein SpaN	pcnB-WT	-0.75612	3.72E-05
44	QCC34422.1	EKN05_024765	IpgB1	protein IpgB	pcnB-WT	-0.73834	1.43E-06
45	QCC34318.1	EKN05_023785	VirF	AraC family invasion system transcriptional regulator VirF	pcnB-WT	-0.7358	0.000119
46	QCC34457.1	EKN05_024260	Shf	carbohydrate transporter	pcnB-WT	-0.71294	0.000314
47	QCC34349.1	EKN05_024155	CopB	replication regulatory protein RepA	pcnB-WT	-0.7129	8.85E-06
48	QCC34405.1	EKN05_024670	Spa15	surface presentation of antigens protein SpaK	pcnB-WT	-0.70287	2.82E-06
49	QCC34385.1	EKN05_024455	PhoN1	phosphatase PAP2 family protein	pcnB-WT	-0.70108	7.01E-05
50	QCC34365.1	EKN05_024250	VirK	virulence factor VirK	pcnB-WT	-0.68041	8.18E-06
51	QCC34386.1	EKN05_024485	UshA	bifunctional UDP-sugar hydrolase/5'-nucleotidase	pcnB-WT	-0.65656	3.98E-06
52	QCC34397.1	EKN05_024630	Spa40	EscU/YscU/HrcU family type III secretion system export apparatus switch prote	pcnB-WT	-0.6022	0.000141
53	QCC34381.1	EKN05_024435	StbA	plasmid stabilization protein	pcnB-WT	-0.59277	5.7E-06
54	QCC34306.1	EKN05_023685	SepA	serine protease autotransporter toxin SepA	pcnB-WT	-0.58351	0.000955
55	QCC34382.1	EKN05_024440	StbB	plasmid stabilization protein	pcnB-WT	-0.53482	0.003843
56	QCC34366.1	EKN05_024255	RfbU	glycosyltransferase family 1 protein	pcnB-WT	-0.50967	0.000286
57	QCC34370.1	EKN05_024305	OspG	protein kinase OspG	pcnB-WT	-0.50855	0.002885

Table R1. Less abundant proteins located on pINV in the *Shigella* Δ*pcnB* vs wt. adj_pvalue ≤0.01; Proteins with Log_2FC ≥1 shown in dark pink; Log_2FC ≥0.5 shown in pale pink.

5. Related to the previous point, in the Materials and Methods section, the methodology for proteomics-based mass spectrometry is clearly described. Nonetheless, the number of independent biological replicates used to generate the proteomics data presented in Figure 2B-C and Table S1 is not explicitly stated. Could you please specify how many biological replicates were analysed for each strain (wt, ΔpcnB, ΔvirF) in this experiment? This information is important to assess the robustness and statistical support of the differential expression analysis.

Response: Thanks for pointing this out. We have used 4 independent biological replicates for each strain for the proteomic profiling. We have now specified this in the Materials and Methods section (Lines 497-498).

6. In Figure 2B and 2C, the volcano plots clearly show the differentially expressed proteins in the proteomic analysis, and the caption confirms that each point represents a protein. However, labels in the plot use names that start with a lowercase letter, a typical convention for bacterial gene names. Since the analysis is based on protein abundance and Table S1 provides data for each "coding gene", there may be a potential inconsistency between the data represented (proteins) and the labeling convention used (genes). Could the authors provide clarification on the choice of this labeling convention or, if it is protein data, consider updating the labels to reflect the standard protein naming convention (first letter capitalized)?

Response: When mapping the peptides from MS analysis we use gene annotations coming from genome assemblies, which are usually better annotated than protein databases. We have now updated the nomenclature in the graphs (Fig 2B-C) and in Table S1 to reflect the standard protein nomenclature.

Reviewer #2:

1. The introduction section is not properly referenced. One major problem is the citation of review articles, as opposed to original articles. For instance, and this is only one example among too many, when referring to VirF as a major virulence factor on line 69, citing Ref #10 is misleading and inconsiderate. Another major problem is the exactness of citations. For instance, Ref #20 does not cover the information presented from lines 91-94 (again, just one example). The authors are therefore invited to review all the information covered in the introduction section and amend the references appropriately.

Response: We have revised the Introduction section, according to the reviewer suggestion.

2. It was difficult to understand how the experiments reported in Fig. 1A and 1D have been conducted. The referenced paper (Di Martino et al, provisionally accepted) is not published and there is no related information in the M&M section. This constant reference to Di Martino et al, provisionally accepted, gets increasingly problematic across the entire paper, because the authors are referring to data that are not accessible to the readers and therefore cannot be evaluated. This needs to be addressed according to the ethical standards of the publication process.

Response: We did in fact provide the related manuscript (Di Martino et al, provisionally accepted) during submission to facilitate the review process, according to PLOS Pathogens submission instructions. We are sorry to hear that the related manuscript did not reach the reviewers alongside the current manuscript. We can now, in either case, report that in June

2025 the related manuscript was published (Di Martino et al, 2025 – Nature Genetics - PMID: 40506541) under full open access. Therefore, any unclarities on this point should hopefully be resolved. We have updated manuscript passages mentioning this other work accordingly.

3. Since the role of pcnB in Shigella virulence plasmid maintenance has been suggested already (PMID: 40424556), the authors do not need Fig 1A/D as introductory materials, and could start the results section with Fig 1B and 1D as confirmatory investigation of existing and published information. Also, please associate Ref #32 to the published PLOS Pathogens paper, not the BioRxiv version.

Response: While finalizing our manuscript for original submission, we came across a preprint, recently published in PLOS Pathogens (12), that investigates the role of *pcnB* (PAP-I) in pathogenic bacteria. This study primarily focuses on *Yersinia* virulence plasmid maintenance, but also includes a smaller dataset on *Shigella* (Fig7A–C therein), where it was observed that a *Shigella ΔipaH2.5ΔpcnB* mutant secretes reduced levels of the IpaB, IpaC, and IpaD virulence proteins. The authors speculate that *pcnB* may promote *Shigella* pINV maintenance. However, the implications for *Shigella* infection and fitness remained to be elucidated. We have aimed to acknowledge and appropriately reference these findings in our original manuscript and we appreciate the opportunity to clarify this point and highlight this reference.

After careful consideration, we believe that retaining Fig. 1A and 1D in our manuscript is important. These data represent the foundational observations that motivated our study. Including them strengthens the reproducibility and robustness of both our study and the recently published PLOS Pathogen paper (12), especially given their complementary but independently derived findings. While our work and the PLOS Pathogens paper start from different angles, together they highlight a broader role for *pcnB* in supporting virulence across enterobacterial pathogens.

Finally, we have updated Reference #32 (now #44) to cite the final published version of the PLOS Pathogens article (PMID: 40424556) instead of the earlier BioRxiv preprint.

4. In Fig 1B, please indicate the growth temperature of bacteria used for generating the input consortium.

Response: Individual bacterial cultures were grown at 30°C before preparing the mix of the input consortium. We have now added this information in the Materials and Methods section (Line 474).

5. Table S1. It is difficult to understand why the log2 FC for pcnB is only -1.5 when comparing a pcnB mutant, which presumably lacks the PCNB protein, to wt? Same problem with VirF.

Response: This is an artifact of using TMT labelling and an MS2 analysis, which stems from peptide co-isolation and from the multiplex nature of these assays (13). Briefly, because the quadrupole cannot isolate very narrow mass windows prior to peptide fragmentation, there are always small "contaminations" from other peptides that co-elute with the identified peptides and end up contributing to the TMT label signal. Thus, it is very difficult to get a zero intensity even when peptides are absent from a sample. This actually serves as a good quality control, since we can see that the "levels" of the deleted gene are among the most downregulated proteins. To avoid confusion, we have now removed these values from the table and the plots and explained that we did so in the methods section (Lines 515-516). We have also updated the text to reflect the revised numbers for less abundant proteins. (Line169,176, 178).

6. Line 177: IcsA is not an actin nucleator. It is a bacterial adaptor that recruits the nucleation promoting factor N-WASP, which then recruits and activates the host cell actin nucleator, ARP 2/3. Please correct.

Response: Amended (Line 173).

7. Fig. 4. The blots are apparently missing in panel B (at least in the PDF version provided to this reviewer).

Response: We are sorry to hear that the Northern blot panels were missing. After reviewing the original submission files, we did not identify any obvious issues at our end. Nevertheless, we hope that the reviewer is now able to fully examine these panels in the final version of the figures.

8. Fig. 5. Although the impact of pcnB on plasmid copy number and T3SS protein expression across the manuscript is rather modest (2-3 fold range), the phenotype observed in enteroids with regard to intracellular infection overtime is rather impressive. This begs the questions as to what is happening to copy number in that system. Previous work has shown that plasmid copy number increases dramatically in vivo during Yersinia infection (PMID: 27365311). Similarly, the authors apparently have the tools to look into copy number in enteroids using qRT-PCR. That would be a fantastic addition to this work.

Response: We agree with Reviewer #2 that assessing plasmid copy number during *Shigella* enteroid infection would be a valuable addition to the study. However, there are technical limitations that currently hinder this experimental effort. Specifically, our infection protocol involves the infection of ~800 organoids (approximately 800,000 cells) at an MOI of 40 for 6 hours. These conditions are optimized to maximize the intracellular bacterial population while minimizing host cell death (14). Despite this, we typically recover only 20,000–100,000 bacteria per infection. This rather small bacterial yield poses a significant challenge for genomic DNA extraction suitable for accurate plasmid copy number quantification. Since the primary focus of this manuscript is to evaluate the impact of *pcnB* on *Shigella* virulence, we consider this a promising direction for future studies, but which we deem non-feasible at this stage.

Nevertheless, it is interesting to note that in *Yersinia pseudotubercolosis*, locking the virulence plasmid (pYV) at three copies was sufficient to restore full virulence in a mouse infection model, in contrast to strains harboring only a single pYV copy (15). This observation aligns with our results and underscores the biological significance of modest changes in plasmid copy number on infection outcomes.

Importantly, this comment prompted us to revisit our live imaging experiments, where we used a plasmid-based reporter (p*uhpt*-GFP) to monitor bacterial spread (4, 16). During this process, we realized that such plasmid reporter system could potentially itself be affected by the loss of *pcnB*, leading to an impaired experimental readout**.** Therefore, to confirm and complement the live imaging findings, we have repeated the *Shigella* infections in epithelial organoid-derived monolayers and performed immunostaining using a polyclonal antibody against *Shigella*. At 12 hours post-infection, we again observed only minimal spread of the Δ*pcnB* mutant within the intestinal epithelium, reinforcing the conclusion from the reporter experiments, namely that the *pcnB* gene promotes *Shigella* intraepithelial expansion (see Fig 6D-E).

9. In general, the discussion section reads like a review of the literature with distant, direct relevance to the topic of the paper.

Response: We have revised the Discussion section, according to the reviewer suggestion.

Reviewer #3:

4. Insufficient detail is given regarding the strains used in the barcoded competition assays.

Response: We have now included additional details regarding the construction of the bacterial strains used in the barcoded competition assays to clarify the experimental design.

5. It is stated that the delta mxiD strain would express virulence genes in the absence of a functional TTSS. While some virulence genes will be expressed, expression of the entire suite of Shigella virulence genes will not be like that of WT if the TTSS is not functional. Functional secretion itself is a significant regulator of virulence gene expression in Shigella.

Response: We appreciate the reviewer's comment. It is indeed correct that full expression of the *Shigella* virulence program depends on a functional Type III Secretion System (TTSS) (17). However, we would like to clarify that TTSS-mediated secretion is only activated in the presence of host cells (18). Therefore, under the experimental conditions used in the barcoded competition assays in Fig 1 B-C (i.e. growth in LB medium), where host cells are absent, secretion is minimal or absent even in the wild-type strain (19). As such, the difference in TTSS functionality between the wild-type and Δ*mxiD* strains is unlikely to affect virulence gene expression in this context. Consistent with this, wt and Δ*mxiD* strains exhibit comparable fitness at both 30°C and 37°C (Fig 1C). We have adjusted the text for clarity (Lines 133-134).

6. It is assumed that organoids were incubated at 37C following infection, but this key information is not provided in the methods section.

Response: This is correct. Organoids were incubated at 37°C following infection. We have now added this information (Line 463).

7. Statistical analyses are needed in Fig. 2A, Fig. 3C, Fig. 4C, Fig. 4D

Response:

We acknowledge that statistical analyses were not included in the originally submitted versions of the mentioned figures. For Fig 3C, which presents previously published RNA-seq data, we have now added the corresponding statistical analysis that was performed in the original study (14). For the remaining figures, we initially considered statistical analysis not strictly necessary, as the data were either supported by complementary experimental evidence (e.g., Fig 2A is corroborated by the proteomic profiling shown in Fig 3B–D) or confirmed previously published findings (e.g., Fig 4C–D reproduce results from earlier work (6)). Nevertheless, we agree that including statistical analysis enhances the clarity and rigor of the presentation, and we have now incorporated the appropriate statistical evaluations into these figures.

We thank the reviewers for their constructive feedback, which has helped us improve the quality of the manuscript. In addition to addressing the reviewer comments, we have carefully pruned the text for clarity and consistency, and ensured compliance with the journal's formatting requirements. We hope that this revised manuscript version will now be suitable for publication in PLOS Pathogens.

The Authors

REFERENCES

1. Pilla G, McVicker G, Tang CM. 2017. Genetic plasticity of the Shigella virulence plasmid is mediated by intra- and inter-molecular events between insertion sequences. PLoS Genet 13.
2. Pilla G, Tang CM. 2018. Going around in circles: virulence plasmids in enteric pathogens. Nat Rev Microbiol 1.
3. Schuch R, Maurelli AT. 1997. Virulence plasmid instability in Shigella flexneri 2a is induced by virulence gene expression. Infect Immun 65:3686–3692.
4. Geiser P, Di Martino ML, Lopes ACC, Bergholtz A, Sundbom M, Skogar M, Graf W, Björner K, Vessby J, Webb D-L, Hellström PM, Eriksson J, Sellin ME. 2025. Determinants of divergent *Salmonella* and *Shigella* epithelial colonization strategies resolved in human enteroids and colonoids. MBio.
5. Nordström K. 2006. Plasmid R1--replication and its control. Plasmid 55:1–26.
6. Söderbom F, Binnie U, Masters M, Wagner EGH. 1997. Regulation of plasmid R1 replication: PcnB and RNase E expedite the decay of the antisense RNA, CopA. Mol Microbiol 26:493–504.
7. Söderbom F, Wagner EGH. 1998. Degradation pathway of CopA, the antisense RNA that controls replication of plasmid R1. Microbiology 144 (Pt 7):1907–1917.
8. De La Cueva-Méndez G, Pimentel B. 2007. Gene and cell survival: lessons from prokaryotic plasmid R1 R1 architecture: a winning design for DNA survival. EMBO Rep 8:458–464.
9. Cao GJ, Sarkar N. 1992. Identification of the gene for an Escherichia coli poly(A) polymerase. Proc Natl Acad Sci 89:10380–10384.
10. Maes A, Gracia C, Innocenti N, Zhang K, Aurell E, Hajnsdorf E. 2017. Landscape of RNA polyadenylation in E. coli. Nucleic Acids Res 45:2746–2756.
11. Celli J, Lesser C, Murphy ER, Blocker AJ, Mattock E. 2017. How Do the Virulence Factors of Shigella Work Together to Cause Disease? Cell Infect Microbiol 7:643389–64.
12. Schubert K, Zhang J, Muscolo ME, Braly M, McCausland JW, Lam HN, Hug K, Loven M, Ruiz Solis S, Estrada Escobar M, Moore H, Terciano D, Fernandez Pacheco D, Lowe TM, Lesser CF, Jacobs-Wagner C, Wang H, Auerbuch V. 2025. The polyadenylase PAPI is required for virulence plasmid maintenance in pathogenic bacteria. PLOS Pathog 21:e1012655.
13. Savitski MM, Mathieson T, Zinn N, Sweetman G, Doce C, Becher I, Pachl F, Kuster B, Bantscheff M. 2013. Measuring and managing ratio compression for accurate iTRAQ/TMT quantification. J Proteome Res 12:3586–3598.
14. Di Martino ML, Jenniches L, Bhetwal A, Eriksson J, Lopes ACC, Ntokaki A, Pasqua M, Sundbom M, Skogar M, Graf W, Webb DL, Hellström PM, Mateus A, Barquist L, Sellin ME. 2025. A scalable gut epithelial organoid model reveals the genome-wide colonization landscape of a human-adapted pathogen. Nat Genet 2025 577 57:1730–1741.
15. Wang H, Avican K, Fahlgren A, Erttmann SF, Nuss AM, Dersch P, Fallman M, Edgren T, Wolf-Watz H. 2016. Increased plasmid copy number is essential for Yersinia T3SS function and virulence. Science 353:492–5.

16. Hausmann A, Böck D, Geiser P, Berthold DL, Fattinger SA, Furter M, Bouman JA, Barthel-Scherrer M, Lang CM, Bakkeren E, Kolinko I, Diard M, Bumann D, Slack E, Regoes RR, Pilhofer M, Sellin ME, Hardt W-D. 2020. Intestinal epithelial NAIP/NLRC4 restricts systemic dissemination of the adapted pathogen Salmonella Typhimurium due to site-specific bacterial PAMP expression. Mucosal Immunol 13:530–544.
17. Le Gall T, Mavris M, Martino MC, Bernardini ML, Denamur E, Parsot C. 2005. Analysis of virulence plasmid gene expression defines three classes of effectors in the type III secretion system of Shigella flexneri. Microbiology 151:951–962.
18. Ménard R, Sansonetti P, Parsot C. 1994. The secretion of the Shigella flexneri Ipa invasins is activated by epithelial cells and controlled by IpaB and IpaD. EMBO J 13:5293–5302.
19. Parsot C, Ménard R, Gounon P, Sansonetti PJ. 1995. Enhanced secretion through the Shigella flexneri Mxi-Spa translocon leads to assembly of extracellular proteins into macromolecular structures. Mol Microbiol 16:291–300.
